# Renal water transport in health and disease

**DOI:** 10.1007/s00424-022-02712-9

**Published:** 2022-06-09

**Authors:** Eric Feraille, Ali Sassi, Valérie Olivier, Grégoire Arnoux, Pierre-Yves Martin

**Affiliations:** 1 Department of Cell Physiology and Metabolism, University of Geneva, 1 Rue Michel Servet, CH-1211 Geneva 4, Switzerland; 2 Service of Nephrology, Department of Internal Medicine, University Hospitals of Geneva, Geneva, Switzerland

**Keywords:** Aquaporin, Diabetes insipidus, Hyponatremia, Kidney tubule, Water balance

## Abstract

Saving body water by optimal reabsorption of water filtered by the kidney leading to excretion of urine with concentrations of solutes largely above that of plasma allowed vertebrate species to leave the aquatic environment to live on solid ground. Filtered water is reabsorbed for 70% and 20% by proximal tubules and thin descending limbs of Henle, respectively. These two nephron segments express the water channel aquaporin-1 located along both apical and basolateral membranes. In the proximal tubule, the paracellular pathway accounts for at least 30% of water reabsorption, and the tight-junction core protein claudin-2 plays a key role in this permeability. The ascending limb of Henle and the distal convoluted tubule are impermeant to water and are responsible for urine dilution. The water balance is adjusted along the collecting system, i.e. connecting tubule and the collecting duct, under the control of arginine-vasopressin (AVP). AVP is synthesized by the hypothalamus and released in response to an increase in extracellular osmolality or stimulation of baroreceptors by decreased blood pressure. In response to AVP, aquaporin-2 water channels stored in subapical intracellular vesicles are translocated to the apical plasma membrane and raise the water permeability of the collecting system. The basolateral step of water reabsorption is mediated by aquaporin-3 and -4, which are constitutively expressed. Drugs targeting water transport include classical diuretics, which primarily inhibit sodium transport; the new class of SGLT2 inhibitors, which promotes osmotic diuresis and the non-peptidic antagonists of the V2 receptor, which are pure aquaretic drugs. Disturbed water balance includes diabetes insipidus and hyponatremias. Diabetes insipidus is characterized by polyuria and polydipsia. It is either related to a deficit in AVP secretion called central diabetes insipidus that can be treated by AVP analogs or to a peripheral defect in AVP response called nephrogenic diabetes insipidus. Diabetes insipidus can be either of genetic origin or acquired. Hyponatremia is a common disorder most often related to free water excess relying on overstimulated or inappropriate AVP secretion. The assessment of blood volume is key for the diagnosis and treatment of hyponatremia, which can be classified as hypo-, eu-, or hypervolemic.

Water is essential for life and is abundantly supplied in an aquatic environment. Adaptation to a terrestrial life required the acquisition of water-saving mechanisms to cope with intermittent water supply. In terrestrial vertebrates such as birds and mammals, this is achieved by the ability of the kidney to reabsorb large amounts of filtered water and to concentrate urine largely above plasma. In humans, the kidneys filter about 180 l each day to excrete about 1–2 l of urine whose osmolality is comprised of between 100 when the water supply is overabundant and 1200 mOsm/Kg H_2_O when the water supply is severely restricted. In this review, we will discuss the physiological control of water balance and renal water handling. We will then summarize our current knowledge on disturbed water balance and drugs increasing renal water excretion.

## Water homeostasis

### Control of water balance

Under physiological conditions, water balance is achieved by equilibration between water gain via food, beverage and metabolism, and water losses via respiration, transpiration, and urine excretion. Water balance is controlled on the one hand by thirst and on the other hand by urinary output.

Thirst is controlled by several groups of neurons located in the lamina terminalis adjacent to the third ventricle and more precisely in the subfornical organ and the organum vasculosum of the lamina terminalis [2, 86]. These neurons, which control both thirst and sodium appetite are sensitive to extracellular osmolality and angiotensin II [29, 42]. For more details, the neural control of thirst has been recently reviewed in depth [8, 97].

Urinary output is dependent on the one hand of the glomerular filtration rate, which determines the amounts of fluid entering the kidney tubules and on the other hand on water reabsorption along the kidney tubules. The glomerular filtration rate is supposed to be fairly constant via tight autoregulatory mechanisms. The water reabsorption process is mostly performed along the proximal tubule and the thin descending limb of the loop of Henle, which reabsorb about 70% and 20% of the filtered water, respectively. Water reabsorption in these two nephron segments is fairly constant under physiological conditions. Water balance is achieved more distally by regulated water reabsorption along the collecting system, i.e. connecting tubule and collecting duct [41].

### Mechanism of vasopressin secretion and action

**Fig. 1:**
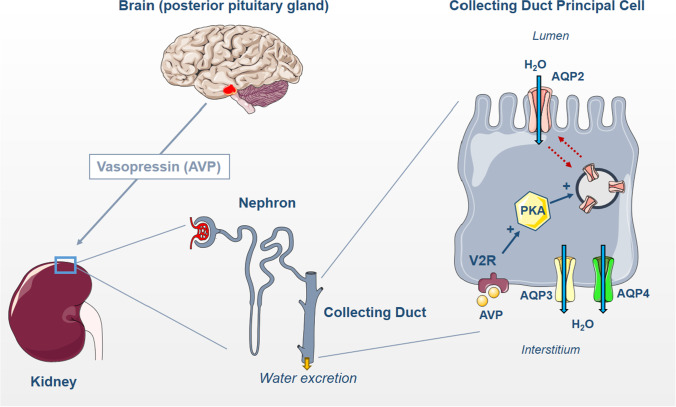
Hormonal control of water transport by collecting duct. Arginine vasopressin (AVP) is synthesized in the hypothalamus and released by the posterior pituitary gland. In the renal collecting duct principal cells, AVP binds to the vasopressin receptor type 2 (V2R), which leads to the activation of the protein kinase A (PKA) and increases aquaporin-2 (AQP2) abundance in the apical plasma membrane by stimulating its translocation from intracellular storage vesicles. Water is reabsorbed via AQP2 at the apical membrane and then exits the cell through aquaporin-3 (AQP3) and aquaporin-4 (AQP4) at the basolateral membrane

Arginine-vasopressin (AVP) also called antidiuretic hormone (ADH) is of seminal importance in controlling water balance through regulation of water reabsorption along the collecting system and thereby urinary output. AVP is a small peptide composed of 9 amino acids. It is synthesized as a large preprohormone by magnocellular neurons of the hypothalamic paraventricular and supraoptic nuclei. AVP is generated in equimolar amounts with neurophysin and copeptin, which are co-secreted at the level of the posterior pituitary gland. AVP is stored in secretory vesicles located at the end of the axons of magnocellular neurons. AVP has a very short half-life (a few minutes) and circulates at a very low concentration (pM range) in plasma. Therefore, the measurement of plasma levels of copeptin, which is much more stable, is used as a surrogate of AVP secretion [24]. This information is summarized in Fig. 1.

AVP secretion is mainly controlled by extracellular osmolality. Increased extracellular osmolality leads to cell shrinkage that is sensed by both AVP-secreting cells and other osmosensitive neurons leading to increased AVP secretion. This osmotic AVP release can be suppressed by anticipation after oral absorption of water, which suppresses the activity of osmosensitive neurons located in the subfornical organ [68]. The so-called osmotic release of AVP is very sensitive and small variations in osmolality of the extracellular fluid are associated with parallel changes in AVP secretion. The threshold of AVP secretion is generally close to 290–295 mOsm/Kg H_2_O [68]. TRPV1 and TRPV4 channels were strongly suspected to mediate osmosensing. However, recent studies in knockout animals did not reveal abnormal AVP secretion in TRPV1 knockout mice [88] or detected slightly increased AVP secretion in response to hypertonic challenge in TRPV4 knockout mice [55], indicating that other important players are at work in this process and remain to be discovered.

Non-osmotic AVP release via high-pressure baroreceptors is a second major stimulus of AVP secretion [79]. Physiological studies have shown that hypotension increases the sensitivity of AVP release in response to extracellular osmolality with a leftward shift of the dose–response curve [28]. Several other stimuli of AVP secretion have been identified such as emotional stress [28] and angiotensin-II [9].

Once released into the bloodstream, AVP reaches its targets and binds to either V1 or V2 type G-protein-coupled receptors. V1 receptors are coupled to calcium signaling and mediate the vasoconstrictor effect of AVP. V2 receptors are expressed by kidney tubule epithelial cells in both medullary and cortical thick ascending limb of Henle, distal convoluted tubule, connecting tubule, and collecting duct (principal cells and inner medullary collecting duct cells) [57] with the highest expression levels in medullary thick ascending limb and collecting duct. In connecting tubule and collecting ducts, V2 receptors are directly involved in the stimulation of water transport [5]. AVP also stimulates sodium transport in the thick ascending limb of Henle, distal convoluted tubule, and collecting system [26]. The binding of AVP to V2 receptors leads to Gαs-dependent activation of adenyl cyclase, generation of cyclic AMP, and activation of protein kinase A, which mediate both short- and long-term effects of AVP [5, 41]. This information is summarized in Fig. 1.

The apelin gene encodes a 77 amino-acid prepropeptide that is subsequently cleaved to generate proapelin and then bioactive peptides (apelin-13, -17, and -36) [34]. Apelin is widely expressed in the central nervous system as well as in peripheral tissues including kidneys. It binds to G-protein coupled receptors (APJ) and inhibits cAMP signaling via Gαi. Both apelin and APJ are expressed in hypothalamic supraoptic and paraventricular nuclei, i.e. the sites of AVP biosynthesis [71]. Inhibition of AVP secretion was shown in response to ventricular apelin infusion [17]. Moreover, physiological studies in humans have shown that extracellular osmolality controls AVP and apelin release in an opposite manner [4]. These studies suggest that apelin physiologically inhibits AVP secretion by the posterior pituitary gland. Apelin also antagonizes the effect of AVP on kidney tubule epithelial cells by the inhibition of cAMP generation [35].

### Kidney tubule water transport and aquaporins

**Fig. 2:**
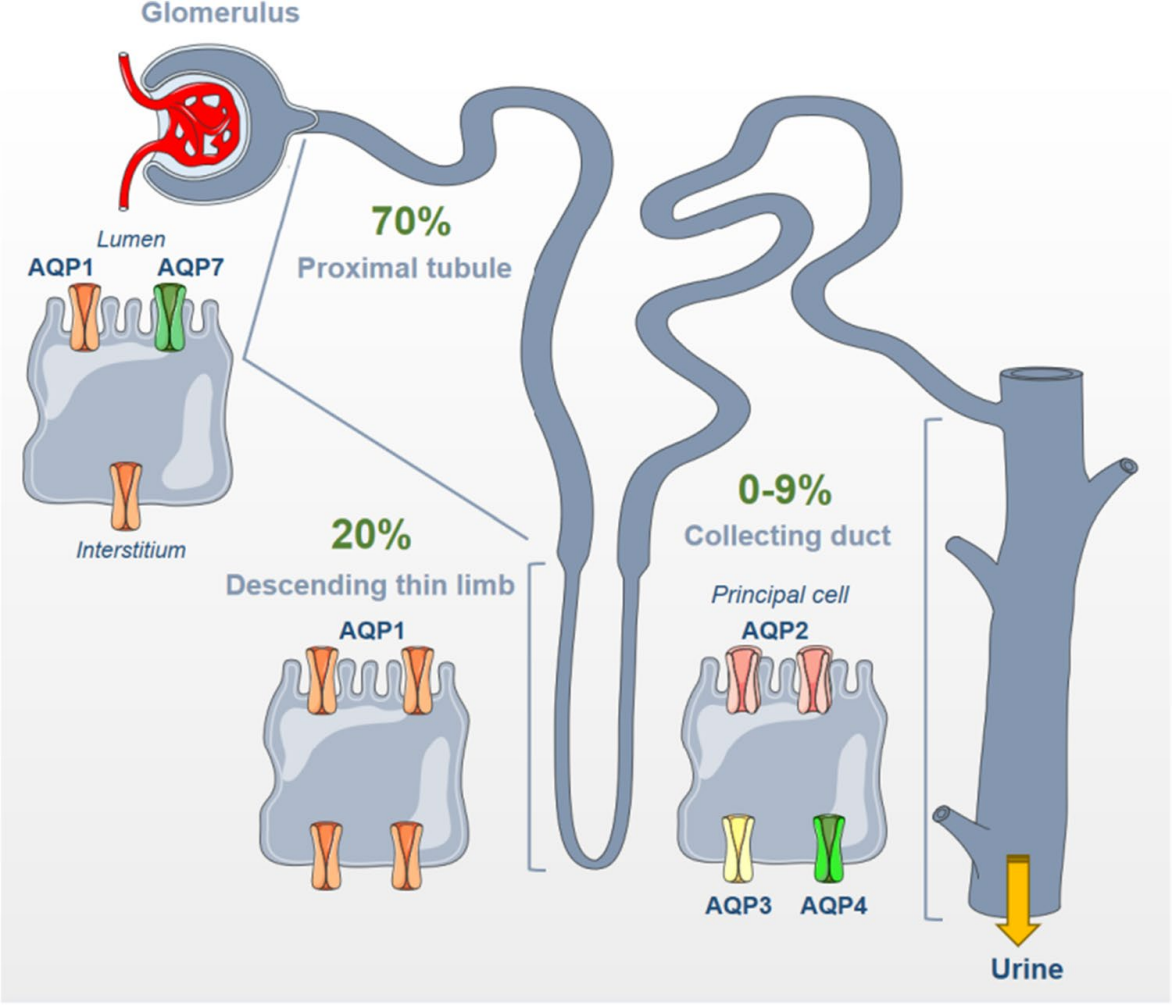
Aquaporin (AQP) distribution along the nephron. AQP1 is located in both the apical and basolateral membrane of proximal tubule cells, which is highly permeable to water and accounts for 70% of total water reabsorption. AQP7 is additionally found along the brush border of proximal tubule cells. The thin descending limb of Henle accounts for 20% of water reabsorption via the AQP1 located at both apical and basolateral sides of the plasma membrane. The collecting duct is less permeable to water (0 to 9%) and expresses three aquaporins in principal cells: AQP2 at the apical membrane, and AQP3 and AQP4 at the basolateral membrane

Water filtered by the glomeruli is efficiently reabsorbed along the kidney tubule. The water permeability of the kidney tubule displays a strong segment-specificity. The proximal tubule is highly water permeable, followed by the thin descending limb of Henle. These two segments are constitutively permeable to water. The thin ascending limb of Henle, the thick ascending limb of Henle, and the distal convoluted tubule are impermeable to water. Finally, the water permeability of the connecting tubule and collecting duct is highly variable and depends mostly on the circulating AVP concentration. This information is summarized in Fig. 2.

#### Water transport by proximal tubule

About 70% of filtered water is reabsorbed along the proximal tubule [26]. This water reabsorption is iso-osmotic and occurs through both transcellular and paracellular pathways. The prototypic water channel aquaporin-1 (AQP1) first identified in red blood cells [69] was subsequently found in the proximal tubule [18, 96]. This AQP1 water channel is located at a high density along both apical and basolateral membranes with little if any intracellular storage [61]. Subsequent functional studies by in situ micropuncture and in vitro microperfusion performed in knockout animals confirmed that AQP1 mediates transcellular water reabsorption in proximal tubules [77, 78]. This transcellular water reabsorption accounts for at least 50% of water transport [77]. Remarkably, proximal tubule luminal hypotonicity was observed in AQP1 knockout mice indicating that transcellular water transport is required for the iso-osmotic reabsorption process in this nephron segment [90]. Aquaporin-7 (AQP7) is a second aquaporin specie found along the brush border of proximal tubule cells [82]. Water permeability of proximal tubules from AQP7 knockout mice is slightly decreased, but these mice do not display obvious water loss or urinary concentration defect [80].

Vectorial water flux is driven by the osmotic gradient generated between the cytosol and the extracellular space comprised of between the deep invaginations of the basolateral plasma membrane of proximal tubule cells [26]. The basolateral Na,K-ATPase actively extrudes intracellular sodium ions that accumulate into the extracellular space, driving anion reabsorption (bicarbonate and chloride) through secondary active (cotransporters) or passive (channels) pathways. This increased osmotic pressure generated by the accumulation of sodium salts is further enhanced by the reabsorption of other solutes such as phosphate, glucose, and amino acids and drives cellular water to the interstitium.

The paracellular pathway may account for at least 25–30% of water reabsorption by the proximal tubule. Paracellular water reabsorption is largely dependent on the expression of the tight-junction core component protein claudin-2 [58, 78], which generates a water-permeable pore [75]. The slight osmotic gradient generated by transcellular solute reabsorption provides the driving force for paracellular water flux from the tubular lumen to the kidney interstitium.

It is worth mentioning that AQP1/claudin-2 double knockout mice do not display a massive reduction in proximal tubule water reabsorption when measured by in vivo micropuncture [78]. Pathways accounting for this residual water reabsorption in proximal tubule from AQP1/claudin-2 double knockout mice remain to be identified.

The water reabsorption process is terminated by the transfer of interstitial fluid to peritubular capillaries. This absorptive process is driven by a very low capillary hydrostatic pressure due to the dissipation of hydrostatic pressure by the afferent and efferent glomerular arterioles and the high capillary oncotic pressure generated by the extraction of “deproteinated” glomerular filtrate from plasma during the glomerular filtration process.

#### Water transport by the thin descending limb of Henle

The thin descending limb of Henle, which plays a major role in the concentration process of the tubular fluid, is impermeable to solutes and highly permeable to water [76]. It constitutively reabsorbs about 20% of the filtered water load. This segment expresses high levels of AQP1 water channels located along apical and basolateral membranes of both short and long thin descending limbs of Henle in one study [61] or only long thin descending limbs in a second one [95]. In vitro micropuncture studies comparing wild-type and AQP1 knockout mice revealed that AQP1 is mandatory for water permeability in the thin descending limb [13].

The hypertonic interstitium generated by the counter-current concentration-dilution process provides the driving force for transcellular water reabsorption. Interstitial water is then reabsorbed by the ascending vasa recta to reach the bloodstream. It is noteworthy to mention that a high water permeability of descending vasa recta is generated by the expression of AQP1 by endothelial cells [65]. This unique property of ascending vasa recta prevents the dilution of the cortico-papillary osmotic gradient via efficient water reabsorption.

#### Water transport by the collecting duct

The collecting system including the connecting tubule and the collecting duct (CD) is the site of regulated water reabsorption. This regulation is performed on the last 10% of the filtered water load resulting in minimal or maximal urine excretion of approximately 0.5 l or 10 l, respectively. Water is specifically reabsorbed by principal cells, which also reabsorb sodium and secrete potassium. It is known for decades that the water permeability of the collecting duct is highly regulated by AVP. In the absence of AVP, the water permeability of the CD is very low under basal conditions, and it rises sharply in the presence of the hormone [56]. It is important to mention that in the presence of AVP, water reabsorption is much larger in the cortical than in the medullary portion of the collecting system. Indeed, the water-free NaCl reabsorption by the thick ascending limb of Henle and the distal convoluted tubule strongly dilutes the tubular fluid. At the end of the distal tubule, the osmolality of the tubular fluid is close to 50 mOsm/KgH_2_O, while interstitial osmolality is close to 300 mOsm/KgH_2_O. This steep osmotic gradient provides the driving force for massive water reabsorption in the presence of AVP. Water subtraction increases intratubular osmolality that would reach a maximum of 300 mOsm/l in the absence of the counter-current concentration process generated by the loop of Henle and the vasa-recta. The cortico-papillary osmotic gradient from 300 to 1200 mOsm/KgH_2_O in humans allows further water subtraction and maximal concentration of the urine up to the maximal interstitial osmolality of 1200 mOsm/KgH_2_O reaching the tip of the papilla. The maximal interstitial osmolality at the tip of the papilla (1200 mOsm/KgH_2_O) can be reached in the presence of AVP, which firstly enhances the active salt reabsorption by the thick ascending limb via stimulation of NKCC2 and secondly, stimulates massive urea reabsorption via activation of urea transporters UT-A1/3 in the inner medullary collecting duct [5]. In addition to the direct stimulation of CD water permeability, AVP also stimulates ENaC and thereby transepithelial sodium reabsorption. This vectorial transport of solutes from the lumen to the interstitium increases the local osmolality, especially inside basolateral plasma membrane infoldings of principal cells, and further enhances water reabsorption [54]. The same process is observed in response to aldosterone, which primarily stimulates sodium reabsorption [25].

The increase in water permeability of the collecting system in response to AVP was first correlated with the appearance of apical membrane protein aggregates [45], suggesting the apical translocation of water pores. Aquaporin-2 (AQP2) was then identified as a collecting duct-specific water channel located in the apical membrane of CD cells [27]. Thereafter, aquaporin-3 (AQP3) [21] and aquaporin-4 (AQP4) [36] were identified as basolateral water channels in CD cells. These three aquaporins are expressed in CD principal cells as well as inner medullary collecting duct (IMCD) cells.

The generation of knockout mice confirmed the key role of AQP2 in water reabsorption since total knockout is lethal within a few days of life [74] and inducible knockout results in severe polyuria [93]. The lethality of the total AQP2 knockout is explained by the absence of compensation by increased AQP2 expression by the CNT [74], which actively participates in water reabsorption as demonstrated by the increased diuresis and decreased concentration capacity of mice with a connecting tubule-specific AQP2 deletion [44]. The functional importance of aquaporins located in the basolateral membrane of CD cells was confirmed by the generation of knockout mice. AQP4 [48] or AQP3 [47] knockout mice displayed a mild phenotype with urinary concentration defect while double knockout was characterized by polyuria [47]. The results confirmed that both aquaporins are expressed along the kidney tubule and partially compensate for each other.

AVP was shown to induce the rapid translocation of subapical intracellular vesicles containing AQP2 to the apical membrane of CD principal cells and to increase the water permeability of the CD [19, 60]. Animal studies suggested that after cessation of the AVP challenge, apical AQP2 undergoes endocytosis and intracellular retrieval [10, 49]. Studies performed in cultured renal epithelial cells (MDCK and LLC-PK cells) transfected with AQP2 showed that a large fraction of AQP2 can be recycled to participate in a new round of translocation to the apical membrane in response to a new AVP challenge [40], while another fraction of AQP2 is ubiquitinated and degraded vis the lysosomal pathway [37]. As recently reviewed in detail [64], at least five AQP2 phosphorylation sites have been identified and were shown to play a role in the translocation and stabilization of the water channel to the apical membrane or its association with the ubiquitination and endocytic types of machinery. The so-called “shuttle hypothesis” implying the recycling of AQP2 for several rounds of intracellular storage and translocation remains to be formally demonstrated in vivo.

AQP2 is transcriptionally controlled by AVP, as demonstrated by the induction of AQP2 expression by administration of AVP to Brattleboro rats, which exhibit a natural *AVP* gene knockout [70] and incubation of cultured CD principal cells in the presence of AVP [32]. This basal AVP-dependent expression of AQP2 is modulated by many other factors among which aldosterone and osmolality are the most physiologically relevant [31, 82]. Aldosterone exerts a biphasic effect on AQP2 expression levels by cultured CD principal cells. After an initial decrease in both AQP2 mRNA and protein levels, aldosterone stabilizes AQP2 protein leading to its accumulation, which generates a synergistic effect with AVP [33]. This effect might be especially important to maintain plasma osmolality under conditions of hyperaldosteronism. The TonEBP-dependent stimulation of *AQP2* transcription in response to extracellular hypertonicity [30] plays an important role to help water reabsorption in the hypertonic kidney medulla. AQP3 expression is also stimulated by long-term AVP challenge although to a lesser extent than AQP2 [84] and is remarkably aldosterone-dependent [46]. Increased AQP4 expression in response to AVP has been shown in the kidney cortex but not in the hypertonic medulla [67]. This observation suggests that hypertonicity inhibits the effect of AVP or that AVP and hypertonicity increase AQP4 expression levels through the same signaling pathway.

## Drugs targeting renal water handling

### Diuretics

Classical diuretics are primarily natriuretic drugs specifically targeting an apical sodium transporter along the kidney tubule. Their pharmacology and mode of action were reviewed in detail elsewhere [22]. Carbonic anhydrase inhibitors, a class of diuretics mostly targeting the proximal tubule are no longer used, except for a few specific indications such as glaucoma or high altitude disease. The so-called “loop diuretics,” including the widely used furosemide, are the most potent diuretics. They inhibit the apical Na/K/2Cl transporter NKCC2, which accounts for 15–20% of tubular sodium reabsorption and is mandatory for the generation of the cortico-papillary osmotic gradient. Therefore, this class of diuretics severely decreases the concentration/dilution power of the kidney. Thiazide diuretics, which inhibit the Na/Cl-cotransporter NCC expressed in the distal convoluted tubule, potentially inhibit up to 5–7% of the reabsorption of filtered sodium. Finally, potassium-sparing diuretics either targeting ENaC either directly or indirectly via blockade or mineralocorticoid receptor, inhibit the reabsorption of 1–5% of the filtered sodium. Diuretics primarily induce natriuresis and secondarily water diuresis via inhibition of urine dilution in the loop of Henle, the distal convoluted tubule, or the collecting system. This effect increases luminal osmolality in the collecting system that decreases the lumen to interstitium osmotic gradient and prevents in part water reabsorption along the connecting tubule and cortical collecting duct. Inhibitors of ENaC also prevent the generation of hypertonicity in the interstitial space between interdigitations of the basolateral membrane of CD principal cells, which drives part of the transcellular water flux [54].

### Inhibitors of SGLT2

The recently developed SGLT2 inhibitors induce an osmotic diuresis by combining glycosuria, natriuresis, and aquaresis. It has been suggested that the metabolic and cardiovascular benefits of SGLT2 inhibitors are, at least, partly explained by the stimulation of water-conserving processes via AVP secretion, partly counterbalancing the osmotic diuresis in CD [52]. AVP not only increases water reabsorption but also facilitates urea transport by IMCD cells and induces accumulation in the inner medulla [7] that contributes to the osmotic driving force for water reabsorption [6]. Moreover, it has been shown that rats treated with SGLT2 inhibitors displayed higher expression levels of the urea transporter UT-A1, suggesting urea-dependent compensatory water reabsorption in the medulla in response to this drug [3, 12]. This urea-driven water conservation process not only relies on the renal urea-recycling pathway but also on the hepatic urea cycle, which generates urea from dietary proteins or from endogenous protein reservoirs. The urea-driven water conservation process induced by SGLT2 inhibitors to maintain water balance increases the metabolic demand and results in a negative energy balance, which participates in the beneficial effects of this drug [59, 98].

#### Non-peptidic inhibitors of the V2 receptor

The non-peptidic inhibitor of the V2 receptor tolvaptan represents a new class of drugs called aquaretics that specifically increase free water excretion. As already mentioned, the availability of these inhibitors has been a long-awaited step to study, understand, and treat hypo-osmolar states. Since the early 1990s, this class of drugs named vaptans has been developed in two subclasses: V1/V2 receptors inhibitors and V2 receptors inhibitors [91]. Four compounds were developed and did exhibit a significant effect on increasing free water excretion and achieving maximal urine dilution. Their efficacy to reverse hyponatremia was demonstrated by clinical studies in the acute setting, and the risks of overcorrection were evaluated as very low. Tolvaptan was also demonstrated to slow the renal cysts growth and to slow the decline in renal function in autosomal dominant polycystic kidney disease [87]. It should be mentioned that the chronic utilization of vaptans was associated with increased risks of hepatotoxicity, a severe side effect that strongly limited their clinical use.

## Disturbed water balance

### Diabetes insipidus

When the AVP pathway does not function correctly, it leads to inadequate urine concentration (hypotonic urine), which is defined as diabetes insipidus (DI). DI is clinically characterized by polyuria/polydipsia and biologically by hyperosmolarity and hypernatremia. DI is classified into two major groups: central (CDI) and nephrogenic (NDI). Another class of DI is gestational DI, which is secondary to very high levels of vasopressinase during pregnancy, which degrades almost instantly circulating AVP [20]. CDI might be genetic or acquired. When associated with *AVP* gene mutation (autosomal dominant (90%) or recessive), the onset of DI is early but gradual, between the first and the sixth year of life [73]. Diagnosis might be difficult according to the pattern of fluid intake. Water deprivation test, which demonstrates the defect in urine concentration ability, can be difficult to interpret, and delayed diagnosis is not unusual. To improve the accuracy of the water deprivation test, AVP can be measured by radioimmunoassay. However, copeptin, which is processed by the same precursor peptide and regulated by the same physiological process, is the easiest to analyze by copeptin assay [16]. Acquired CDI has several causes, which include tumors, cranial trauma, post-neurosurgery operations, and infections. The symptoms may vary in severity and can be transient. In adults, the water deprivation test is easiest to perform and interpret than in children. Primary (dipsogenic) polydipsia is one differential diagnosis, which can be excluded. Neuroradiology, especially MRI is of utmost importance to determine the cause of acquired CDI. Fortunately, substitutive treatments are available in addition to free access to water. Synthetic AVP analogs with a longer half-life than AVP (desmopressin) are available by different routes: oral, nasal spray, sublingual, or parenteral [15].

Nephrogenic diabetes insipidus (NDI) is characterized by a normal physiologic release of AVP in response to an increase in plasma osmolarity, which, however, does not translate into subsequent hypertonic urine. Therefore, “nephrogenic” determines a disrupted cellular pathway of the principal cells going from V2 receptors (V2R) mutations (majority of cases of genetic NDI) to water channels (AQP2) dysfunctions (majority of acquired NDI). The disease (especially the *X*-linked recessive mutations of AVP2R) starts at birth and delayed diagnosis is frequent. As the polyuria is more severe, newborns might suffer from the cerebral sequela of chronic dehydration and mental development is frequently suboptimal and mortality more elevated [14]. Acquired NDI is better identified especially when associated with drug toxicity such as lithium, electrolytes disorders (hypokalemia, hypercalcemia), or ureteral obstruction. Usually, the concentration power alteration is less severe and not as debilitating as in CDI. However, as production of AVP is not the problem, desmopressin has no place (almost) in the treatment, and appropriate fluid intake is the hallmark of treatment. Some drugs, such as NSAIDs and thiazides diuretics, improve the symptoms by enhancing cellular response to AVP and also, for hydrochlorothiazide, by decreasing water and sodium delivery to the collecting tubules. This effect of thiazides most likely relies on a stimulated proximal sodium and water reabsorption. Other drugs have been demonstrated to stimulate AQP2 migration to the luminal membrane by a cAMP-dependent mechanism such as PGE2 and rolupram or by AVP- and cAMP-independent mechanisms such as simvastatin, sildenafil, and metformin. These drugs might be promising when combined to mitigate the concentrating defect [53]. Targeting AQP2 regulation is the most promising therapeutic development. Several approaches are investigated including direct stimulation of cAMP generation, the major activator of AQP2, inhibition of cAMP phosphodiesterase, stimulation of AQP2 phosphorylation by cGMP kinase… [63]. It should be emphasized that DI patients have usually normal plasma osmolality providing a normal thirst mechanism and adequate access to fluid.

### Hyponatremia

**Fig. 3:**
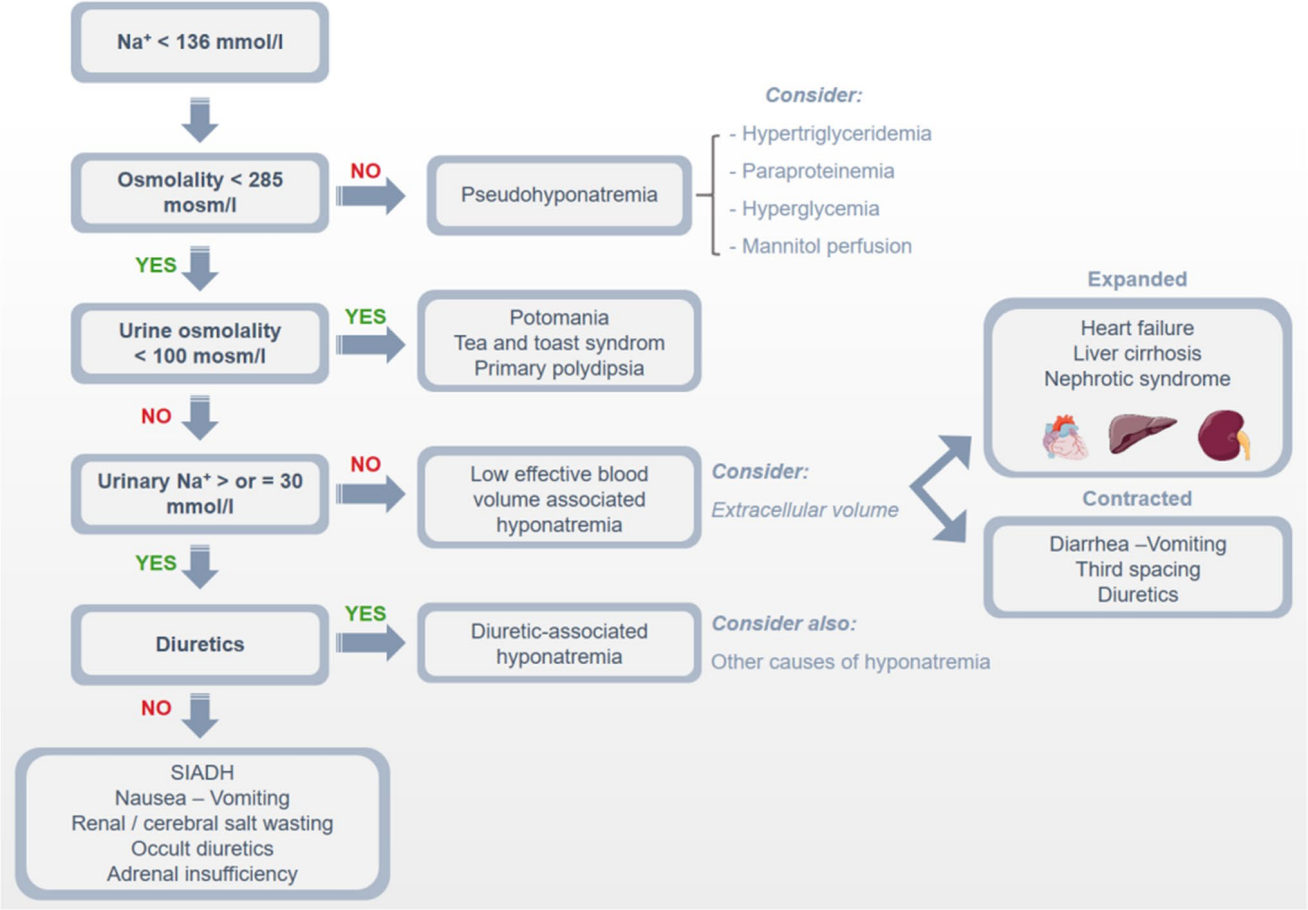
Major biological and clinical characteristics of the various types of hyponatremias

Hyponatremia (plasma sodium concentration less than 135 mmol/L) is considered the most frequent electrolyte disorder. It should be, however, reminded that hyponatremia is most often caused by a defect in free water excretion, which results in an increased plasma water/sodium plasma ratio rather than by sodium loss. This defect in free water excretion has been demonstrated at a molecular level in situations of hyponatremia such as cardiac failure, wherein animal models and in humans, inappropriate activation of AQP2 has been observed [51, 62]. The term “hypo-osmolar state” would be more appropriate as it would allow discriminating this disorder from other electrolytes disorders such as hypo-, hyperkaliemia, hypo-hypercalcemia, etc.…, which indicate, in most cases, loss or excess of the electrolyte incriminated. The most important task when plasma sodium is decreased is to determine the extracellular volume status. Indeed, hyponatremia can be seen in a state of hypervolemia, euvolemia, and hypovolemia and are universally classified as hyponatremia hypo-eu-or hypervolemic [85]. Therefore, the accuracy of extracellular assessment is necessary to properly classify and treat hyponatremia (Fig. 3). Evaluation of volume status is not always straightforward. When the hyper-or hypovolemia is mild, clinical signs are scarce. Diagnosis should combine careful medical history taking, physical examination, and laboratory parameters analyses. Amongst those, urine electrolytes and osmolarity are of utmost importance. Collecting urine samples simultaneously with blood samples is the best (and only) way to estimate how the kidney is operating in the setting of hyponatremia. When urinary osmolarity is higher than plasma osmolarity, this demonstrates that AVP is not appropriately suppressed to inhibit free water reabsorption and produce hypotonic urine. In addition, sodium fractional excretion (and/or urea excretion) gives an important clue to the extracellular volume management by the kidney. Other laboratory parameters such as glucose (to exclude pseudohyponatremia), urea, creatinine, K, Cl, total bicarbonates, and urates are extremely useful for integrative analyses. Recently, point of care (POC) cardiac ultrasound has been demonstrated to improve physical examination in the diagnosis of hyponatremia [11]. A proper classification related to extracellular volume status is important because the consequences of hyponatremia differ depending on this classification. Finally, the timeline and the severity of hyponatremia have to be determined. European and American guidelines [81, 92] defined natremia between 135 and 130 as mild, 129–125 as moderate, and < 125 as severe or profound. Similarly, acute hyponatremia is defined when documented in less than 48 h, whereas chronic hyponatremia exists for more than 48 h. When timing is not possible, chronic is the default choice unless clinical symptoms are very severe. These elements demonstrate that hyponatremia cannot be only considered an isolated laboratory parameter but necessitates a comprehensive and systematic clinical approach.

For more than 40 years, studies have demonstrated the prognostic values of hyponatremia to assess the severity of the disease in the setting of cardiac failure [83], liver failure, pneumonia, hip fractures, etc. Recent studies confirmed this early observation and indicate that hyponatremia is a severity marker of diseases such as stroke [66], frailty [39], and pre-end-stage kidney disease [50]. These findings demonstrate that hyponatremia has to be considered a risk factor and has to be identified as such. A major issue is the clinical relevance of hyponatremia as a modifiable parameter, which is associated with a better outcome. To summarize many studies, the prognosis is improved when the underlining cause of hyponatremia is successfully treated (cardiac failure, liver failure, pneumonia, etc.) leading to the correction of hyponatremia but not modified by treating hyponatremia per se. The availability of non-peptide V2R antagonists (vaptans) was a major step to address this issue. Their roles in the long-term management of major diseases such as cardiac failure or liver cirrhosis are still a matter of debate. In patients with cardiac failure, the administration of tolvaptan did improve hyponatremia, but it did not modify the outcomes at 24 months [43]. However, post hoc analyses did identify the class of patients who had an improved outcome with tolvaptan [89]. In liver cirrhosis, tolvaptan may improve long-term survival if the therapeutic response is sustained in Japanese patients [38], but other studies did not observe this better outcome, and EASL does not recommend its use for long term [1], whereas the Japan Society of Hepatology is more in favor [94]. More studies are therefore needed to better delineate the long-term use of tolvaptan.

Interventional studies were performed in hypervolemic hyponatremia where the non-osmotic release of AVP is triggered by baroreceptors-induced stimuli secondary to organ dysfunction. This situation is not transposable to hypovolemic or euvolemic hyponatremia such as syndrome of the inappropriate release of AVP (SIADH). In the first situation, AVP release has another trigger than osmolarity but is appropriate due to strong hemodynamic stimuli, whereas, in the second situation (SIADH), AVP is in excess because of dysregulation of its secretion or ectopic release. This second situation might be in certain circumstances deleterious by itself and might have clinical consequences. For instance, in patients with mild or moderate hyponatremia, an increased incidence in falls secondary to balance/gait troubles induced by hyponatremia has been demonstrated [72]. Up to now, no randomized studies have been performed to demonstrate that correction of chronic hyponatremia might decrease the risk of falls. However, a recent study suggests that tolvaptan decreases hyponatremia symptoms such as unsteady gait, dizziness, and confusion [23]*.*

## Abbreviations

AQP: Aquaporin
AVP: Arginine-AVP
CD: Collecting duct
CDI: Central diabetes insipidus
DI: Diabetes insipidus
IMCD: Inner medullary collecting duct
CDI: Central diabetes insipidus
NDI: Nephrogenic diabetes insipidus
